# miR‐149 controls non‐alcoholic fatty liver by targeting FGF‐21

**DOI:** 10.1111/jcmm.12848

**Published:** 2016-04-06

**Authors:** Junjie Xiao, Dongchao Lv, Yingying Zhao, Xiaoyu Chen, Meiyi Song, Jingqi Liu, Yihua Bei, Fei Wang, Wenzhuo Yang, Changqing Yang

**Affiliations:** ^1^ Regeneration and Ageing LabExperimental Center of Life SciencesSchool of Life ScienceShanghai UniversityShanghaiChina; ^2^ Innovative Drug Research Center of Shanghai UniversityShanghaiChina; ^3^ Division of Gastroenterology and HepatologyDigestive Disease InstituteTongji HospitalTongji University School of MedicineShanghaiChina

**Keywords:** Non‐alcoholic fatty liver disease, miR‐149, fibroblast growth factor‐21

## Abstract

Non‐alcoholic fatty liver disease (NAFLD), a lipid metabolism disorder characterized by the accumulation of intrahepatic fat, has emerged as a global public health problem. However, its underlying molecular mechanism remains unclear. We previously have found that miR‐149 was elevated in NAFLD induced by high‐fat diet mice model, whereas decreased by a 16‐week running programme. Here, we reported that miR‐149 was increased in HepG2 cells treated with long‐chain fatty acid (FFA). In addition, miR‐149 was able to promote lipogenesis in HepG2 cells in the absence of FFA treatment. Moreover, inhibition of miR‐149 was capable of inhibiting lipogenesis in HepG2 cells in the presence of FFA treatment. Meanwhile, fibroblast growth factor‐21 (FGF‐21) was identified as a target gene of miR‐149, which was demonstrated by the fact that miR‐149 could negatively regulate the protein expression level of FGF‐21, and FGF‐21 was also responsible for the effect of miR‐149 inhibitor in decreasing lipogenesis in HepG2 cells in the presence of FFA treatment. These data implicate that miR‐149 might be a novel therapeutic target for NAFLD.

## Introduction

Non‐alcoholic fatty liver disease (NAFLD), a multi‐factorial metabolism‐related disorder characterized by the accumulation of intrahepatic fat, is the hepatic manifestation of metabolic syndrome and is closely associated with increased cardiovascular diseases‐related mortality 1. Non‐alcoholic fatty liver disease represents one of the most prevalent chronic liver diseases in developed countries caused by complex interactions, except alcohol or other definite causes, which affects at least 25% individuals in the general population 2, 3, 4. The major risk factors that have been established for NAFLD include obesity, diabetes mellitus, hyperlipidaemia, elevated glucose and insulin resistance 2. Non‐alcoholic fatty liver disease may progress from simple steatosis to its more aggressive forms, non‐alcoholic steatohepatitis, which is featured with hepatocyte injury, hepatic inflammation and hepatic fibrosis. Non‐alcoholic steatohepatitis can finally develop to hepatic cirrhosis, liver failure and even hepatocellular carcinoma 5, 6, 7. Although NAFLD has emerged as a global public health problem, its pathogenesis remains unclear, especially steatosis formation and regulation 1, 4. A better understanding of the molecular mechanisms underlying NAFLD will help develop more effective therapeutic interventions for NAFLD 1, 4.

MicroRNAs (miRNAs, miRs) are a class of small short (20–23 nucleotides) non‐coding RNA molecules regulating gene expressions at posttranscriptional level 8. It is estimated that almost 60% of gene expressions were regulated by miRNAs 8, 9, 10, 11. As important regulators for gene expression, miRNAs participate in cell growth, proliferation, differentiation, apoptosis and metabolism 8, 9, 10, 11. Dysregulated miRNAs have been reported to participate in a lot of diseases including cardiovascular diseases, diabetes mellitus and metabolic syndrome 8. Interestingly, the involvement of miRNAs in the development of NAFLD has also been reported 12, 13, 14, 15. These miRNAs include miR‐122, miR‐451, miR‐155, miR‐34a, miR‐33a/b, *etc*. 12, 13, 14, 15. Recently, we reported that miR‐212 was elevated in liver of high‐fat diet (HFD)‐fed mice, and a 16‐week running programme could protect the liver from HFD‐induced liver steatosis with decreased miR‐212 level 16. In addition, miR‐212 also contributes to lipogenesis in HepG2 cells 16. Interestingly, in that study, we also found that miR‐149 was increased in the liver of HFD‐fed mice, whereas exercise could prevent liver steatosis with blunted miR‐149 expression 16. Therefore, in this study, we aimed at investigating the role of miR‐149 in NAFLD based on an *in vitro* model of lipogenesis performed with HepG2 cells treated with long‐chain fatty acid (FFA). We firstly found that miR‐149 was increased in HepG2 cells treated with FFA. Interestingly, miR‐149 was also able to promote lipogenesis in HepG2 cells in the absence of FFA treatment, whereas inhibition of miR‐149 could attenuate that in the presence of FFA treatment. Our data also indicate that fibroblast growth factor‐21 (FGF‐21) is a target gene of miR‐149. Thus, targeting miR‐149 might represent a novel therapeutic strategy for NAFLD.

## Materials and methods

### Cell culture and long‐chain fatty acid treatment

The human hepatoma cell line HepG2 cells were obtained from the Cell Bank of Chinese Academy of Sciences (Shanghai, China), and cultured in high glucose–DMEM (Hyclone, Logan, UT) containing 10% foetal bovine serum (FBS; Hyclone), 100 U/ml penicillin and 100 mg/ml streptomycin. Cells were incubated at 37°C in a humidified incubator with 5% CO_2_.

To establish an *in vitro* cell model of NAFLD, HepG2 cells were exposed to FFA prepared in culture medium containing 1% bovine serum albumin (BSA; Keygen, Nanjing, Jiangsu Province, China) at a final concentration of 1 mM for 24 hrs.

### Cell transfection

miR‐149 mimics (50 nM), miR‐149 inhibitors (100 nM) and their negative controls were purchased from RiboBio (Guangzhou, China). A small interfering RNA (siRNA, 75 nM) for FGF‐21 and a non‐target control siRNA were also obtained from RiboBio. HepG2 cells at 70% confluency were serum starved by 1% FBS containing medium for 6 hrs and transfected with the miRNAs and siRNAs by Lipofectamine 2000 (Invitrogen, Carlsbad, USA) according to the manufacturer's instruction. Twenty‐four hours after transfection, medium was substituted for serum‐free medium containing 1‐mM FFA or 1% BSA, and cells were harvested at 48 hrs for quantitative real‐time reverse transcription‐polymerase chain reactions (qRT‐PCRs), Western blot analysis, nile red staining and flow cytometry.

### qRT‐PCRs

HepG2 cells (1 × 10^6^) were cultured in 12‐well plates and treated differently. Total RNA was isolated from HepG2 cells performed with Trizol (TaKaRa, Dalian, Liaoning Province, China) according to the manufacturer's instruction. The Bulge‐Loop miRNA RT‐PCR (RiboBio) was used for the analysis of miR‐149. U6 was used as a reference control. qRT‐PCRs were performed based on the SYBR Green I method (TaKaRa) in a CFX96^™^ Real‐Time PCR Detection System (Bio‐Rad, Richmond, CA, USA), according to the manufacturer's protocol. The 2^−ΔΔCt^ method was applied to determine the relative miRNA expression levels.

### Western blot analysis

HepG2 cells (1 × 10^6^) were cultured in six‐well plates. Forty‐eight hours after different treatment, cells were washed with ice‐cold PBS and lysed with 200‐μl radioimmunoprecipitation assay (Keygen) buffer with 1‐mM phenylmethanesulfonyl fluoride (Keygen). The cell lysates were scraped and transferred to an EP tube, and clarified by centrifugation at 12,000 × g for 15 min. at 4°C. Protein concentration was measured by a BCA protein assay kit (TaKaRa) according to the manufacturer's instructions. Total protein was separated by 10% SDS‐PAGE (Bio‐Rad Laboratories, Hertfordshire, UK), and transferred to Polyvinylidene Flouride (PVDF) membranes. After blocked with 5% skimmed milk at room temperature for at least 2 hrs, the membranes were probed with the primary antibodies for fibroblast growth factor‐21 (FGF‐21, 1:1000; Millipore, Bedford, MA, USA) and β‐actin (1:10,000; ABclonal, Cambridge, MA, USA) as an internal control at 4°C overnight. The membranes were washed by tris‐buffered saline with 1% Tween for at least three times, followed by incubation with diluted horseradish peroxidase‐conjugated secondary antibody (1:10,000; Bioworld, Dublin, OH, USA) at room temperature for 1 hr. After rinsing, bands were visualized by enhanced chemiluminescence (Tanon, Shanghai, China) with Tanon 5200 chemiluminescence imaging system (Tanon).

### Flow cytometry

Cells were collected with 0.25% trypsin (Gibco, San Diego, CA) by centrifugation at 73 g for 5 min. at room temperature, washed with PBS and fixed in 4% paraformaldehyde for 10 min. Cells were then washed again and stained with nile red dye for 15 min., and analysed with flow cytometer (Beckman, Miami, FL, USA). Fluorescence intensity of HepG2 cells was obtained for calculating the mean value of the control groups. Cell intensity above the mean value was considered as the positive cells and the data were expressed as the fold change comparing to the control group.

### Statistical analysis

The data were expressed as the means ± S.E. A Student's *t*‐test or one‐way anova was conducted with a Bonferroni's *post hoc* test performed with spss version 20.0 (IBM Armonk, New York, USA). Statistical differences were considered to be significant at *P* < 0.05.

## Results

### miR‐149 is up‐regulated in FFA‐treated HepG2 cells

**Figure 1:**
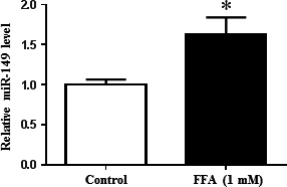
miR‐149 is increased in FFA‐treated HepG2 cells *in vitro*. FFA, long‐chain fatty acid treatment, *P < 0.05.

The *in vitro* cell model of NAFLD was successfully established by exposing HepG2 cells to 1‐mM FFA for 24 hrs as previously reported 16. Based on this NAFLD cell model, we found that miR‐149 was significantly increased (Fig. 1). Interestingly, in our previous study, miR‐149 was also found to be up‐regulated in the liver tissues of NAFLD mice induced by HFD 16, further demonstrating that miR‐149 might contribute to NAFLD.

### miR‐149 regulates lipid droplet formation in HepG2 cells

**Figure 2:**
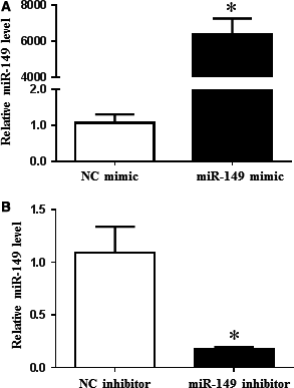
miR‐149 mimic and inhibitor increases and decreases miR‐149 in HepG2 cells, respectively. Quantitative real‐time reverse transcription‐polymerase chain reactions confirmed that miR‐149 mimic (**A**) and inhibitor (**B**) took effect in HepG2 cells, *P < 0.05.

**Figure 3:**
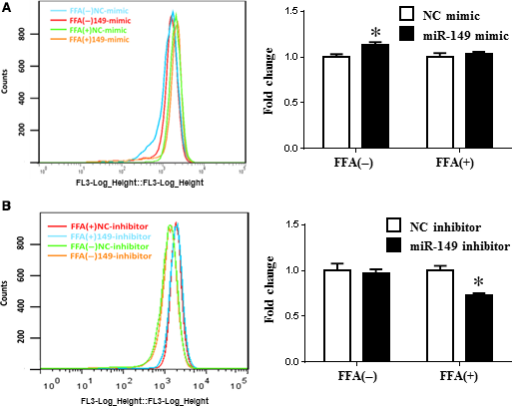
Effect of miR‐149 on lipogenesis in HepG2 cells. Flow cytofluorometry indicated that miR‐149 was able to promote lipogenesis in HepG2 cells in the absence of FFA treatment, whereas it cannot further promote lipogenesis in the presence of FFA treatment (**A**). In addition, inhibition of miR‐149 was capable of attenuating lipogenesis in HepG2 cells in the presence of FFA treatment (**B**). FFA, long‐chain fatty acid treatment, *P < 0.05.

To identify the regulatory effect of miR‐149 on lipid deposit, loss‐of‐function and gain‐of‐function analysis was performed in HepG2 cells with and without FFA treatment. qRT‐PCRs were first conducted to confirm that miR‐149 mimics and inhibitors successfully took effect in HepG2 cells (Fig. 2). miR‐149 was able to promote lipogenesis in HepG2 cells in the absence of FFA treatment, whereas it could not further promote lipogenesis in the presence of FFA treatment (Fig. 3A), indicating that miR‐149 mimics have a positive role in promoting lipid droplet formation when the level of lipid accumulation was low in HepG2 cells. Moreover, inhibition of miR‐149 reduced lipogenesis in HepG2 cells in the presence of FFA treatment (Fig. 3B), indicating that inhibition of miR‐149 was able to suppress lipid droplet formation in the condition of high level of intracellular lipid deposition.

### FGF‐21 is identified as a target gene of miR‐149

**Figure 4:**
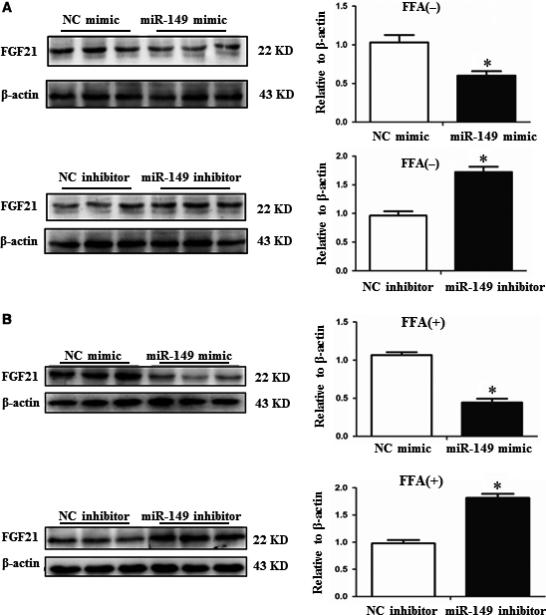
FGF‐21 is negatively regulated by miR‐149. miR‐149 negatively regulates FGF‐21 at protein level in HepG2 cells without FFA (**A**) or with FFA (**B**) treatment. FFA, long‐chain fatty acid treatment, *P < 0.05.

**Figure 5:**
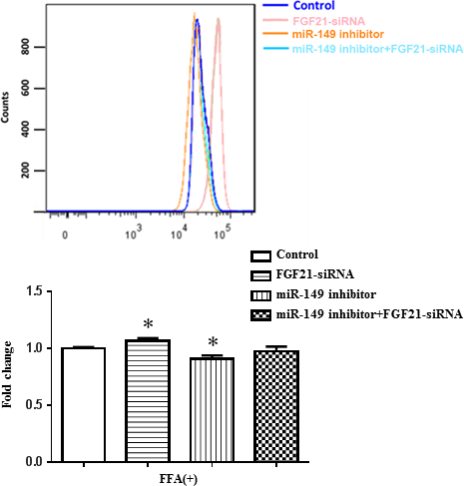
FGF‐21 is a target gene of miR‐149 involved in lipogenesis. Flow cytofluorometry indicated that the FGF‐21 siRNA significantly abolished the inhibitory effect of miR‐149 on lipogenesis in HepG2 cells in the presence of FFA. FFA, long‐chain fatty acid treatment, *P < 0.05.

As shown in miRWalk database, FGF‐21 is a well‐known target gene of miR‐149. Given that FGF‐21 is a key regulator for lipid metabolism 16, we continued to investigate whether FGF‐21 was a target gene of miR‐149 in HepG2 cells. We found that miR‐149 mimics down‐regulated, whereas miR‐149 inhibitors up‐regulated FGF‐21 expression at protein level regardless of FFA treatment (Fig. 4). A FGF‐21 siRNA was used here to further determine whether FGF‐21 was responsible for the effect of miR‐149 in the lipogenesis in HepG2 cells. As determined by flow cytometry, silencing FGF‐21 increased lipid content in HepG2 cells, whereas the inhibitory effect of miR‐149 inhibitor on lipogenesis was prevented by FGF‐21 siRNA in the presence of FFA treatment (Fig. 5). These results indicate that FGF‐21 is a target gene of miR‐149 involved in lipogenesis.

## Discussion

Non‐alcoholic fatty liver disease, characterized by excessive hepatic lipid accumulation, is one of the most common chronic liver diseases 3, 4. A ‘double‐hit’ hypothesis has been interpreted for the development of NAFLD 17. Lipid deposition in the liver is the ‘first hit’, whereas inflammation, hepatocellular injury and fibrosis are the ‘second hit’ 17. Currently, the treatment for NAFLD is not totally satisfactory, although sensible diet, exercise, antioxidants, *etc*. have been used 1, 18, 19. Therefore, novel therapeutics for NAFLD is highly needed 1. Here, we report that miR‐149 is capable of promoting lipogenesis in HepG2 cells in the absence of FFA treatment, whereas inhibition of miR‐149 is able to attenuate that in the presence of FFA treatment. FGF‐21, a target gene of miR‐212 that we have previously reported 16, has been identified as a target gene of miR‐149 as well. Thus, targeting miR‐149/FGF‐21 might represent a novel therapeutic method for NAFLD.

Accumulating evidence suggests that dysregulated miRNAs contribute to NAFLD, such as miR‐451 and miR‐212 12, 13. To date, over 1880 miRNAs have been reported in human according to the records from miRBase 21. By taking advantage of miRNA arrays, we previously identified that miR‐149 was significantly increased in the mouse liver suffered from NAFLD, indicating that up‐regulation of miR‐149 might be a contributor for NAFLD 16. miR‐149 has been reported as a tumour suppressor and could inhibit cell proliferation and cell‐cycle progression in many types of cancers 20, 21, 22. The polymorphisms of miR‐149 have been associated with ischemic stroke, silent brain infarction risk, gastrointestinal cancer risk and myocardial infarction 23, 24, 25. Besides that, miR‐149 can also regulate FGF‐2 signalling and FGF‐2‐induced responses in human endothelial cells including cell proliferation, migration and cord formation 26. However, the role of miR‐149 in liver remains unclear. Previously, we found that miR‐149 was elevated in the liver tissues of NAFLD mice induced by HFD 16. Interestingly, a previous study has reported that skeletal muscles from HFD‐fed mice exhibited down‐regulation of miR‐149 27. This indicates a tissue‐specific role of miR‐149. Importantly, we found that miR‐149 was able to promote lipogenesis in HepG2 cells in the absence of FFA treatment, whereas miR‐149 inhibitors were capable of inhibiting lipogenesis in HepG2 cells in the presence of FFA treatment. To the best of our knowledge, this study firstly indicates the role of miR‐149 in NAFLD.

*FGF‐21* is a member of the broad ligand family of the FGF signalling system, which includes 22 factors in human 28. *FGF‐21* has been implicated in the control of cell survival, tissue repair, energy homeostasis and glucose and lipid metabolism 29. *FGF‐21* has been reported to be highly expressed in liver, and in muscle, pancreas, white adipose tissue and testis as well 30. Interestingly, human FGF‐21 is about 75% identity homologous to mouse FGF‐21, whereas FGF‐21 is less than 35% identify homology with other human FGFs, indicating that FGF‐21 is unique in structure 30. Interestingly, over‐expression or pharmacological administration of FGF‐21 has been shown to ameliorate fatty liver, obesity and type 2 diabetes 30. Thus, FGF‐21 has been increasingly recognized as a promising intervention therapy for metabolic diseases including NAFLD 30. As FGF‐21 is a validated target gene of miR‐149, we confirmed that the inhibitory effect of miR‐149 inhibitors on lipogenesis was blunted in the presence of FFA treatment by silencing FGF‐21. Interestingly, FGF‐21 is also a target gene of miR‐212 in NAFLD that we have previously reported 16. Thus, our data further support that FGF‐21 is a promising target for NAFLD at least.

Several limitations of this study should be highlighted here. Firstly, FGF‐21 has been identified as a target gene of miR‐149 in this study and also a target gene of miR‐212 in our previous study 16. However, the biological function of these two miRNAs is slightly different, indicating that other target genes should be investigated in the future. Secondly, hepatic FGF‐21 expression is regulated by peroxisome proliferator‐activated receptor‐α (PPAR‐α) and FGF‐21 is also able to act as an upstream inducer of peroxisome proliferator‐activated receptor protein‐1α (PGC‐1α) 30. The complex regulatory network between miR‐149 and other molecules including PPAR‐α FGF‐21, and PGC‐1α should be clarified 30. Thirdly, although beyond the scope of this study, it would be interesting to determine if miR‐149 down‐regulation is responsible for the protective effect of exercise in NAFLD in the future.

In conclusion, our study shows that miR‐149 regulates lipogenesis by targeting FGF‐21. Pharmacological inhibition of miR‐149 might be a novel therapeutic strategy for NAFLD.

## Conflict of interest

The authors declare that there are no conflicts of interest.
